# Structure and Diffusion of Ionic PDMS Melts

**DOI:** 10.3390/polym14153070

**Published:** 2022-07-29

**Authors:** Argyrios V. Karatrantos, Jettawat Khantaveramongkol, Martin Kröger

**Affiliations:** 1 Materials Research and Technology, Luxembourg Institute of Science and Technology, 5, Avenue des Hauts-Fourneaux, L-4362 Esch-sur-Alzette, Luxembourg; kj555771@gmail.com; 2 Polymer Physics, Department of Materials, ETH Zurich, Leopold-Ruzicka-Weg 4, CH-8093 Zurich, Switzerland

**Keywords:** polymer electrolytes, ionomers, ionic group distribution, grafted polymers, counterion condensation, ion transport

## Abstract

Ionic polymers exhibit mechanical properties that can be widely tuned upon selectively charging them. However, the correlated structural and dynamical properties underlying the microscopic mechanism remain largely unexplored. Here, we investigate, for the first time, the structure and diffusion of randomly and end-functionalized ionic poly(dimethylsiloxane) (PDMS) melts with negatively charged bromide counterions, by means of atomistic molecular dynamics using a united atom model. In particular, we find that the density of the ionic PDMS melts exceeds the one of their neutral counterpart and increases as the charge density increases. The counterions are condensed to the cationic part of end-functionalized cationic PDMS chains, especially for the higher molecular weights, leading to a slow diffusion inside the melt; the counterions are also correlated more strongly to each other for the end-functionalized PDMS. Temperature has a weak effect on the counterion structure and leads to an Arrhenius type of behavior for the counterion diffusion coefficient. In addition, the charge density of PDMS chains enhances the diffusion of counterions especially at higher temperatures, but hinders PDMS chain dynamics. Neutral PDMS chains are shown to exhibit faster dynamics (diffusion) than ionic PDMS chains. These findings contribute to the theoretical description of the correlations between structure and dynamical properties of ion-containing polymers.

## 1. Introduction

Polydimethylsiloxane (PDMS) is the most widely explored and utilized polysiloxane, due to its extremely low glass transition ($T_{g}=-125$ °C), non-toxicity, excellent thermal stability, high gas permeability, fine optical transparency, oxidative stability, UV resistance and good biocompatibility [1,2]. It is used in biomedicine [3,4], microfluidics [5,6,7], MEMS [8], triboelectricity [9] and piezoelectrics [10]. Motivation to explore the structure and dynamics of ionic PDMS [11,12,13,14,15,16,17,18,19,20,21,22,23,24] or other melts [25,26,27,28,29,30,31,32,33,34,35,36,37,38], is very high since ionic interactions inherit polymers with a functionality that leads to a high performance and applications as gas-separating membranes [15], water purification membranes [13,14,16], energy storage devices [39,40] as well as in sensing [22,23], actuation [21], fuel cells [41], and others [18,19,24,42].

In particular, end-linked PDMS melts, using carboxyl ionomers [43,44,45], reinforced with gallium trivalent cationic counterions have been studied with respect to their mechanical behavior and swelling properties [11]. The morphology and rheology of PDMS ionomers with a varying number of ions per chain, and as a function of the cation type, have been investigated by means of small angle X-ray scattering and scanning electron microscopy techniques. Different morphologies were observed for different cations [12]. For instance, PDMS ionomers containing gallium cations form inhomogeneously distributed polydisperse spherical aggregates, low mol% concentration of zinc and cobalt ionomers do not aggregate, whereas high mol% concentration zinc ionomers exhibit spherical and rodlike aggregates [12]. Nevertheless, the counterion type has little influence on the elastic modulus [12]. The equilibrium state of ionomers is reached rapidly at high temperatures and demonstrates a physically cross-linked network [12,31,32]. Such networks can swell in a non-polar solvent [11], whereas hydrophobic ionic polysiloxanes can be formulated by the incorporation of ionic groups [18,46]. Furthermore, there are very recent experimental efforts that functionalize PDMS chains with tertiary amine N${}^{+}$(CH${}_{3}$)${}_{3}$ group, reinforced with Br${}^{-}$ anions, either on their chain ends (telechelic) or randomly grafted along the backbone (random copolymer) [19]. The ionic functionalization of PDMS increased the viscosity compared to the unmodifield PDMS melts. In contrast to the ionic grafted PDMS melt, the end-functionalized PDMS melts showed a greater propensity for solid-like behavior [19]. Although there are very recent experiments into the rheological characterization of ionic PDMS melts [19], there are no experimental measurements focusing on the density, polymer dimensions, or the ion and polymer dynamics (diffusion) of ionic PDMS melts.

Recently, fully atomistic simulations of ionic polystyrene sulfonate melts with Na${}^{+}$ counterions were implemented [35]. It was found that the placement of the ionic backbone group (sulfonate: SO${}_{3}^{-}$) affects the shape of formatted clusters and the packing of the ionic group inside each cluster, as can be observed from pair correlation functions. In addition, polystyrene mobility is affected by the ionic group contribution and is higher for the block chains than for the random and precise (ionic groups at precise distance) chains [35]. The Na${}^{+}$ counterions condense on the O${}_{3}^{-}$ groups, with faster dynamics observed for block polymers. However, due to the strong electrostatic attraction, diffusion is not achieved during the simulation time span of these studies. Faster counterion dynamics was observed in melts with semiflexible chains than in melts with flexible chains [47]. In addition, the strength of the electrostatic interactions, as characterized by the Bjerrum length $\ell_{B}$, is controlled by the dielectric constant [46] of the polymer medium and temperature. In one coarse-grained simulation study, it was observed that an increase of $\ell_{B}$ led to a sublinear monotonic increase of the glass transition temperature of an ionic melt [48].

Most simulation studies of dry ionomer melts or glasses [29,49] have been based on coarse-grained models that lacked chemical detail [29,47,49,50,51,52,53,54,55] and only studies on ionic polymer melts have been performed with atomistic force fields are scarce [28,56,57,58,59]. In such atomistic simulations, ionic aggregation was observed from the existence of a low wavevector peak (ionomer peak) [60,61,62] in the scattering structure factor of ionomer melts. While the linear size of aggregates can be estimated using scanning transmission electron microscopy (STEM) data, it cannot resolve the shape of aggregates (spherical versus stringlike) which is prone to affect the percolation network and thus, conductivity. However, this issue can be addressed by coarse-grained molecular dynamics which can reveal the aggregate morphology [57,63]. Studies of coarse-grained models revealed that aggregates in systems with randomly sequenced polymers tend to be more stringlike and exhibit a wider size distribution in comparison to systems with periodic charge-spacing [64]. In another recent work, the phase diagram of ionic aggregate types was modeled for random ionomers [65]. At a high electrostatic strength or high ion concentration, percolated ionic aggregates can occur [65]. Counterion dynamics was found to be dependent on the morphology of aggregates, exhibiting a faster diffusion in percolated aggregates [56,66]. This effect has been confirmed by atomistic simulations [67]. Furthermore, ionic aggregates also affect the polymer dynamics due to the presence of the ion cross-linked network [33,68].

To the best of our knowledge, there are no previous simulation studies on diffusion in ionic melts and in particular on the ionic PDMS melts behavior, therefore, we investigate here for the first time the structure and dynamic properties of ionic PDMS melts and their counterions, using a united atom model. The paper is organized as follows. Section 2 introduces the applied methodology and simulation details. Section 3.1 investigates the structure of polymers and ions in the polymer melt. The polymer dimensions of all the melts studied (different polymer charge densities) are reported in Section 3.2. In Section 3.3 we calculate and compare the diffusion of Br${}^{-}$ counterions and PDMS chains for the different ionic polymer architectures. Conclusions are offered in Section 4.

## 2. Methodology

**Figure 1:**
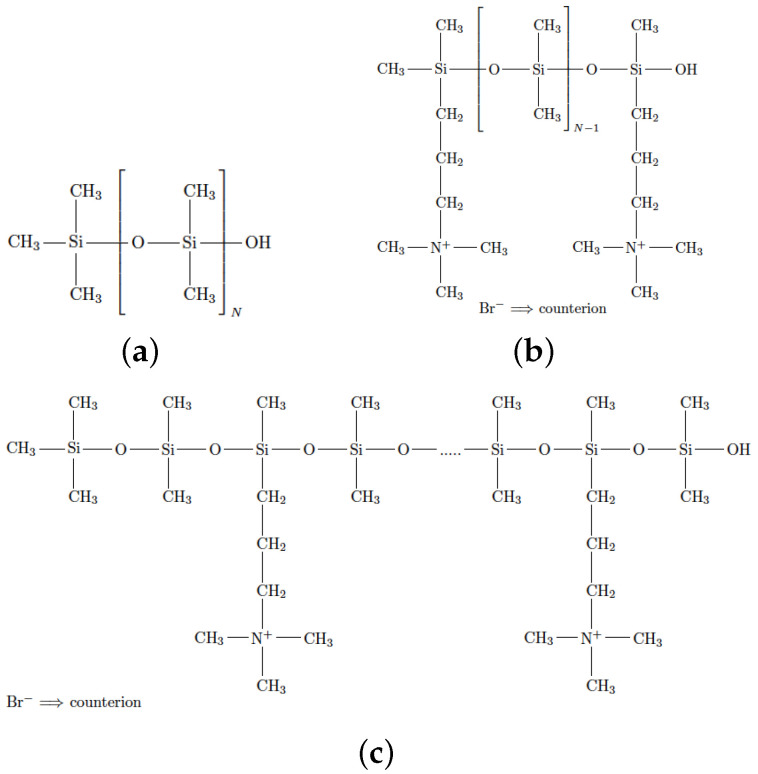
Chemical structures of poly(dimethylsiloxane). (**a**) neutral PDMS chain, (**b**) ionic PDMS chain functionalized on its chain ends, (**c**) ionic PDMS chain functionalized grafted randomly (random copolymer) along its backbone. The bromide (Br${}^{-}$) counterions (anions) are dispersed in the melt.

Our melt systems are composed of neutral or ionic PDMS chains. The ionic chains carry a permanent positive charge, either on the chain ends or randomly grafted along the backbone (random copolymer), as shown in Figure 1. We prepare ionic melts with charged monomer fractions f=2.5%, 5%, and 10%, where *f* is the ratio of functionalized monomers to the total number of monomers (Table 1). We studied unentangled ionic PDMS chains with up to 10% fraction of charged monomers since we have also studied those systems experimentally [19].

The united atom model, which does not incorporate hydrogen atoms, has been used to simulate such PDMS chains and the force field is taken from [69]. The Lennard–Jones parameters for Br${}^{-}$ and N${}^{+}$ atoms were obtained from Refs. [70,71], respectively. The torsion potentials of the grafted oligomer on the PDMS backbone were taken from the TraPPE [72] united atom force field [73,74]. The neutral PDMS chains studied here are not functionalized with hydroxyl groups on their chain ends [75], thus there is no hydrogen bonding interactions between the PDMS chains in the melt [69]. The ionic melts are neutral overall due to the presence of negatively charged bromide (Br${}^{-}$) anions. To account for PDMS chain polarizability [76], we scaled down the charges of nitrogen (N${}^{+}$) and Br${}^{-}$ anions by 50% [77,78,79,80,81,82,83,84]. PDMS melts with two different polymerization degrees, N=40 and N=80, are studied for all three cases illustrated in Figure 1.

The Lorentz–Berthelot mixing rules $\epsilon_{ij}={\left(\epsilon_{i}\epsilon_{j}\right)}^{1/2}$ and $\sigma_{ij}=\left(\sigma_{i}+\sigma_{j}\right)/2$ [85] were employed, specifying the strength and range of the Lennard–Jones type interactions between non-bonded united atoms [86]. In addition, the Coulomb interaction between charged united atoms is incorporated and given by [85]

(1) $$V_{ij}^{\mathrm{Coulomb}}=\frac{q_{i}q_{j}}{4\pi \epsilon_{r}\epsilon_{0}r_{ij}}$$

where $q_{i}$ is charge of atom *i*, and $r_{ij}$ the Euclidean distance between atoms *i* and *j*. The long-range electrostatics was computed using the particle-mesh Ewald method (PME) [87,88] in contrast to the less accurate Reaction field method that was used to model PDMS melts in previous studies [69]. The simulations were performed in a cell starting from dilute concentrations, built with PACKMOL [89] and energy-minimized. Subsequently, using the isothermal isobaric (NPT) ensemble, molecular dynamics simulations at P=1 atm and temperatures T=300 K, 375 K, 425 K, 450 and 473 K were performed for 600–1100 ns, in total. The densities were equilibrated at each temperature. The production runs were 200 ns long. Simulation parameters are summarized in Table 1.

We checked that the linear size of the simulation cell was larger than the root mean square end-to-end distance of the polymer chains. To set the temperature at constant *T* and constant *P*, the Nosé–Hoover thermostat and Parrinello–Rahman barostat were implemented respectively. Their relaxation times were 2 ps and 2 ps, respectively. The leap-frog algorithm [90] was used to update the coordinates and velocities with a time step equal to Δt=1 fs. The initial velocities were generated using a Maxwell–Boltzmann distribution at each temperature. The cutoff distance of the short-range neighbor list [90], van der Waals and Coulombic forces was set at 1.45 nm [69]. Long range dispersion corrections for energy and pressure were applied. The Fourier spacing of PME electrostatics was 0.12 nm, and the PME order was equal to 4 [90]. The molecular dynamics simulations were performed using the GROMACS package [85,90,91,92].

## 3. Results and Discussion

### 3.1. Melt Density and Structure

**Figure 2:**
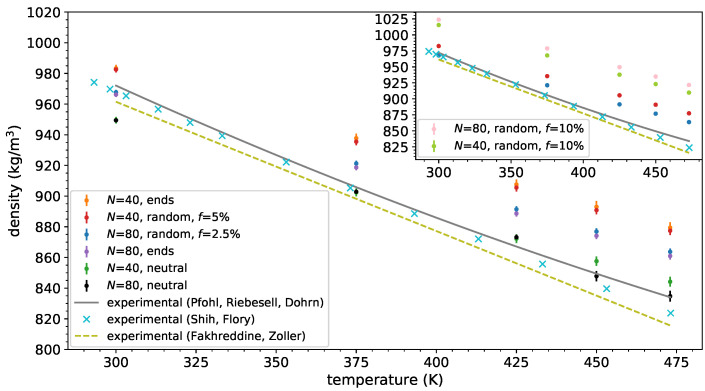
Density of (ionic) PDMS melts by simulations for various *N* and charged monomer fraction *f*, and by experiments. (i) Solid line: experimental values available from [93], (ii) cross symbols, experimental values taken from [94], (iii) dashed line: experimental values by [95], (iv) magenta symbols: end-functionalized N=80, (v) orange symbols: end-functionalized N=40, (vi) red symbols: randomly functionalized N=40, f=5%, (vii) blue symbols: random N=80, f=2.5%, (viii) green symbols: neutral N=40 melt, (ix) black symbols: neutral N=80 melt. Inset: (i) green symbols: random N=40, f=10%, (ii) pink symbols: random N=40, f=10%.

First, we calculated the mass density of the neutral and ionic PDMS melts and depicted them in Figure 2. As can be seen, the united atoms model for PDMS melts yielded a density highly comparable with experimental values for neutral PDMS melts for all temperatures studied, except at the lowest 300 K where the model underpredicts the experimental value. It can also clearly be seen that the end-functionalized ionic PDMS melts exhibit a higher density than for the neutral melts. The charge sequence does not seem to affect the density: The random ionic copolymers and end-functionalized ionic PDMS melts with the same charged monomer fraction exhibit the same density (within the error margin). It can be seen in the inset of Figure 2 that the presence of a higher charged monomer fraction, *f*, along the PDMS backbone leads to an increase in the total density of the system. This result is verified by comparing the random copolymer melts (f=10%) for both molecular weights (N=40,80) with the lower charged random copolymers. By decreasing the molecular weight (N=40) of the ionic PDMS, the density of the system decreases throughout the whole temperature range, due to more chain ends being created and the accompanying increase of free volume.

**Figure 3:**
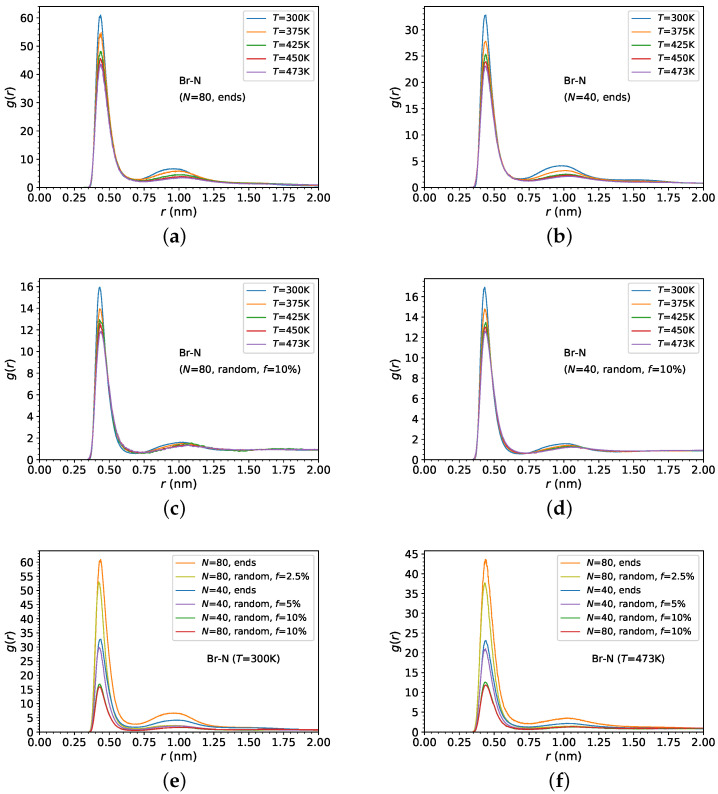
Br${}^{-}$-N${}^{+}$ radial distribution functions $g_{\mathrm{Br},N}\left(r\right)$ of (**a**,**b**) end-functionalized PDMS chains, (**c**,**d**) f=10% randomly grafted functionalized PDMS chains, at different temperatures. (**a**,**c**) N=80 versus (**b**,**d**) N=40 and (**e**,**f**) for various charged monomer fractions at (**e**) T=300 K, (**f**) T=473 K.

In order to explain the differences in the densities of the ionic PDMS melts and investigate their morphology, we focus on the analysis of radial distribution functions (RDFs), or the pair correlation functions, mainly between Br${}^{-}$ anions and between Br${}^{-}$ anions and N${}^{+}$ cations in the functionalization chain of the ionic PDMS (Figure 3). The RDF or radial pair correlation function $g_{\mathrm{AB}}\left(r\right)$ between two atom types A and B is defined by [85,96,97]:

(2) $$g_{\mathrm{AB}}\left(r\right)=\frac{V}{N_{B}N_{A}}\sum_{i}^{N_{A}}\sum_{j}^{N_{B}}\frac{\delta (r_{ij}-r)}{4\pi r^{2}}$$

where *V* is the total system volume, and $N_{A}$ and $N_{B}$ the number of atoms of type A and B, respectively. In other words, g(r) provides the local spatial ordering in the isotropic melt.

In particular, we can see in Figure 3a that there is a strong first peak between Br${}^{-}$ anions and N${}^{+}$ cations on the end-functionalized PDMS; however, temperature has a rather weak effect on the Br–N correlation, demonstrating the stronger correlation at lower temperatures. This shows that Br${}^{-}$ anions condense more strongly on the N${}^{+}$ cation of the functionalized PDMS side chain due to the strong electrostatic attraction (lower Bjerrum length $\ell_{B}$) and the lower ion mobility at lower temperatures. Such an effect has also been observed in polyelectrolyte solutions. In addition, a weaker Br${}^{-}$ anion condensation on the N${}^{+}$ cation of the functionalized side chain appears for lower molecular weight (N=40) PDMS chains, as can be observed in Figure 3b.

**Figure 4:**
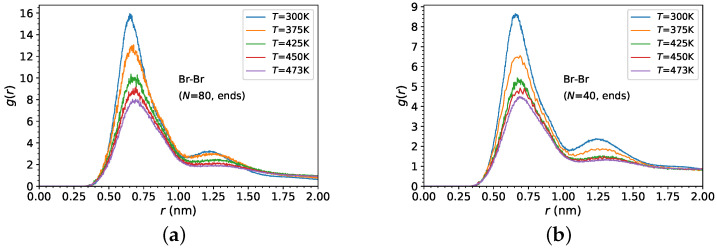

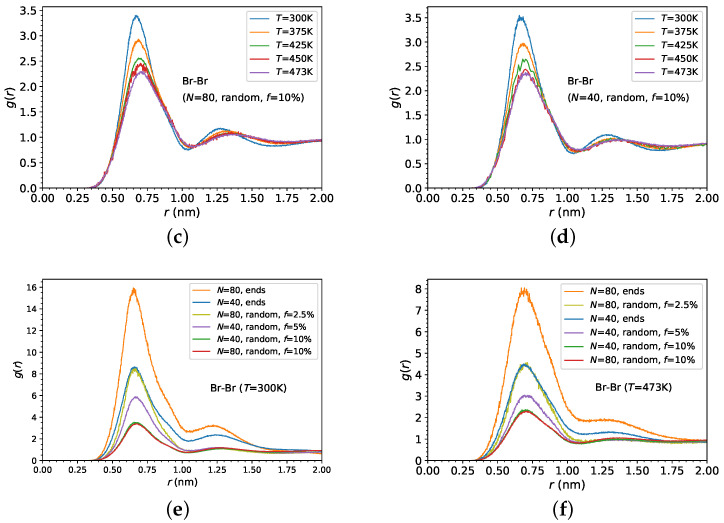
Br${}^{-}$-Br${}^{-}$ radial distribution functions $g_{\mathrm{Br},\mathrm{Br}}\left(r\right)$ of (**a**,**b**) end-functionalized PDMS chains, (**c**,**d**) randomly grafted f=10% functionalized PDMS chains, at different temperatures. (**a**,**c**) N=80 versus (**b**,**d**) N=40 and (**e**,**f**) for various charged monomer fractions *f* at two different temperatures, (**e**) T=300 K and (**f**) T=473 K.

Figure 3c shows the radial distribution function between Br${}^{-}$ anions and N${}^{+}$ cations, in the same temperature range, for random ionic PDMS copolymers. It can be seen as a distinct effect of temperature on the structure of Br${}^{-}$ anions, showing a stronger condensation at lower temperatures. However, the condensation of Br${}^{-}$ anions on the N${}^{+}$ cations is much weaker for the random PDMS copolymers than for the end-functionalized PDMS chains (Figure 3a,b), as is depicted from the height of the first peak of the radial distribution function in Figure 3c,d. In such random copolymers, the molecular weight has a very weak effect on the Br${}^{-}$ anions condensation. Furthermore, Figure 4a–d depict the Br${}^{-}$ anions radial distribution function showing a distinct effect on both first and second coordination cells of ionic PDMS architectures and temperatures.

### 3.2. Polymer Dimensions

**Figure 5:**
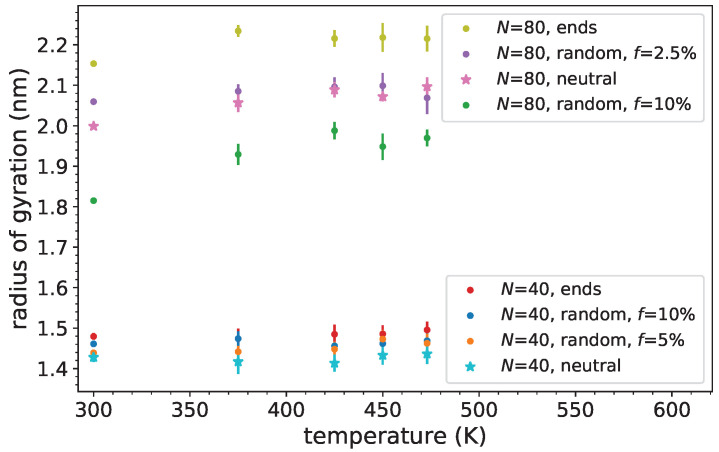
Radius of gyration $R_{g}$ versus temperature *T* of PDMS chains with N=80 and N=40 (neutral, end- and randomly grafted functionalized PDMS chains).

In this section, we focus our attention on the analysis of the PDMS dimensions of all studied melts. In particular, the radius of gyration $R_{g}$ is a measure of average polymer chain dimensions, and is defined as the square root of the mean squared distance between monomers and the center of mass of their chains [85,98],

(3) $$R_{g}^{2}\left(N\right)=\frac{1}{\sum_{i=1}^{N}m_{i}}\langle \sum_{i=1}^{N}m_{i}{\left|r_{i}-r_{\mathrm{cm}}\right|}^{2}\rangle ,$$

where $r_{i}$ is the position of atom *i*, and $r_{\mathrm{cm}}$ is the center of mass of the chain. The average is taken over all chains and the ensemble. We can see in Figure 5 that the PDMS dimensions are affected by the ionic architecture of the PDMS for the higher molecular weight (N=80) polymers. In particular, the $R_{g}$ of end-functionalized PDMS chains is larger than that in the other systems, thus showing that the end-functionalized chains are stretched more, whereas the conformations of ionic random PDMS copolymers, for f=10%, are more collapsed, in comparison to the neutral PDMS chains. In the case of end-functionalized PDMS chains, the Br${}^{-}$ counterions are condensed most strongly on the N${}^{+}$ atoms of the grafted chains (Figure 3e,f), and at the same time they are most strongly correlated to each other (Figure 4e,f), thus making the end-functionalized PDMS chains to stretch more in comparison to random ionic PDMS copolymers with f=10%, where the Br${}^{-}$ counterions are condensed the least on the N${}^{+}$ atoms (Figure 3e,f) and least correlated to each other (Figure 4e,f). In addition, we can see that there is a decrease of the $R_{g}$ of PDMS chains, in all systems with N=80 at low temperatures (T=300 K). Above T=300 K, the $R_{g}$ is not altered by temperature, outside the error margin. Moreover, for low molecular weight (N=40), similarly to chains with N=80, end-functionalized PDMS is more stretched at T=300; however, the ionic architecture of PDMS does not have any significant effect on the $R_{g}$ at higher temperatures.

### 3.3. Ion and Polymer Diffusion

In this section, we measure the translational diffusion coefficients, by molecular dynamics, from the asymptotic behavior of the mean square displacement (MSD) [99,100,101]:

(4) $$D_{0}=\frac{1}{6}\lim_{t\to \infty }\frac{d}{dt}\langle |r_{i}\left(t\right)-r_{i}{\left(0\right)|}^{2}\rangle$$

where $\langle |r_{i}\left(t\right)-r_{i}\left(0\right){|}^{2}\rangle$ is the time dependent MSD of the particles (atoms) of chains, or ions, averaged over time and over the atoms (or ions) of the ensemble.

**Figure 6:**
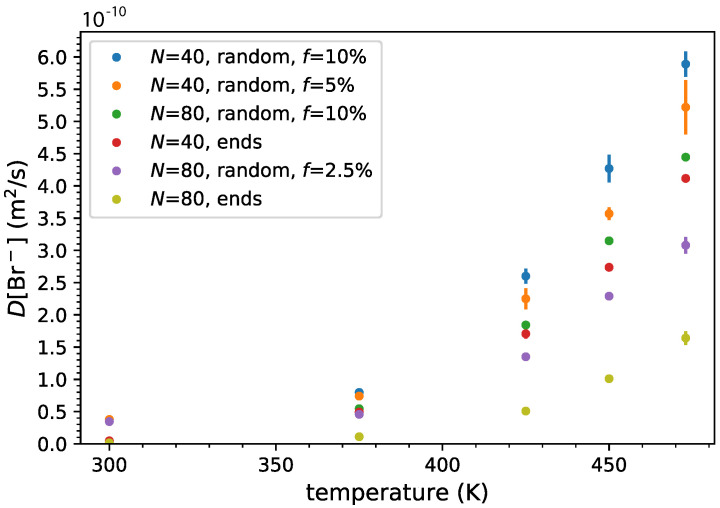
Bromide diffusion coefficient *D* versus temperature for end- and randomly grafted functionalized ionic PDMS melts.

First, we focused on the dynamics of the Br${}^{-}$ anions in the ionic PDMS melts. The mean square displacement of the Br${}^{-}$ anions shows that the linear diffusion regime was reached for temperatures equal to or higher than 300 K, as depicted in Supplementary Figure S1. By fitting the linear regime of the mean square displacement, we calculated the diffusion coefficient of Br${}^{-}$ anions in ionic PDMS melts (Figure 6). At 300 K, the counterions exhibit very slow dynamics. As expected, higher temperatures enhance the dynamics of the counterions following an Arrhenius type of relation, which depends on the ionic charge density and localization of the charges on the PDMS backbone.

In particular, it can be seen that, for both molecular weights, the Br${}^{-}$ anions in the random copolymer melts diffuse faster than the end-functionalized polymers, at all temperatures higher than 300 K. For the higher molecular weight (N=80) of random copolymers, the dynamics of Br${}^{-}$ anions decreases (thus they diffuse slower) [102]. It is worth noting that, although the chain charge density increases the density of the melt, it also enhances the Br${}^{-}$ diffusion.

The presence of ionic functionalization on the backbone also enhances the Br${}^{-}$ diffusion, especially at higher temperatures (T≥450 K). The slowest diffusion appears for the Br${}^{-}$ present in end-functionalized PDMS, in particular those with a high molecular weight (N=80), due to the stronger localization and interaction of the anions on the cationic nitrogen ends.

**Figure 7:**
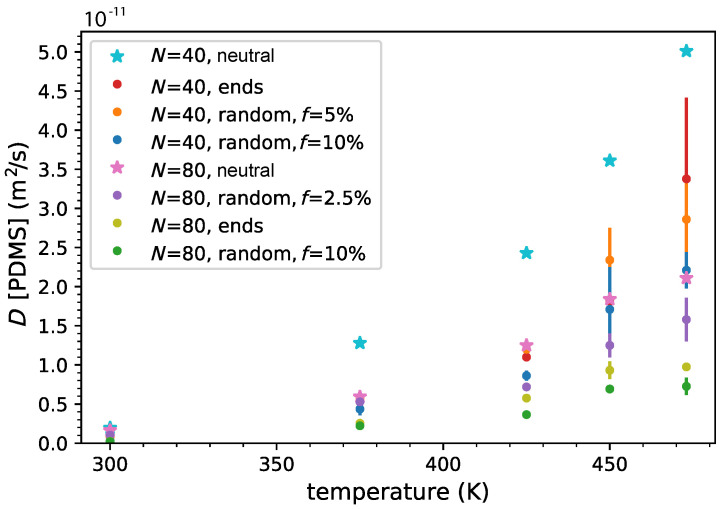
PDMS chain diffusion coefficient versus temperature for end- and randomly grafted functionalized ionic PDMS and neutral PDMS melts.

To obtain an overview of the diffusional behavior, we also calculated the dynamics (diffusion) [100,103] of the PDMS chains (from the MSD of all atoms in a chain) for all the systems studied and depicted in Figure 7. It is worth noting that the calculated PDMS diffusion coefficient in the neutral melt, for N=40 ($M_{w}\approx$ 3 k), is $D=1.39\times 10^{-11}$ m${}^{2}$/s at 375 K, which is of the same order as the experimentally measured, by the pulse field gradient-NMR technique, diffusion coefficient of PDMS chains ($M_{w}\approx$ 3.5 k), $D=2\times 10^{-11}$ m${}^{2}$/s at 323 K [44]. It is also noted that the ionic charges, on the polymer chain, hinder the PDMS chain dynamics (and thus diffusion); thus the chains, at all temperatures, in the neutral melt diffuse faster. As expected, we found that the ionic PDMS chains with a higher molecular weight (N=80) show slower dynamics. The end-functionalized PDMS chains for N=40,80 do not show the slowest dynamics as was observed on the Br${}^{-}$ anions diffusion, but the end functionalization has a rather subtle effect on PDMS dynamics. The charged monomer fraction, *f*, of the random copolymers hinders the ionic PDMS chain dynamics, showing the opposite behavior to that appearing in the Br${}^{-}$ dynamics. In particular, the random PDMS copolymers with f=10% present the slowest diffusion, outside the error margin, whereas the Br${}^{-}$ diffusion is the fastest for N=80 monomers. The diffusion coefficient of PDMS chains is approximately one order of magnitude lower than that of the Br${}^{-}$, as can be seen by comparing Figure 6 and Figure 7, having a lower activation energy than that of the Br${}^{-}$ case. Furthermore, we calculated the apparent transference number $t_{-}$ (when correlations of ion motion are neglected) of the anions using the defining equation $t_{-}=D_{-}/\left(D_{-}+D_{+}\right)=D_{{\mathrm{Br}}^{-}}/\left(D_{{\mathrm{Br}}^{-}}+D_{\mathrm{PDMS}}\right)$ [104]. We obtained an average value of $t_{-}\approx 0.85-0.95$ (Figure S3), showing that the Br${}^{-}$ counterions contribute mainly to the overall conductivity; however, we could not identify any effect of temperature or ionic charge density.

## 4. Conclusions

To summarize, we investigated the density, structure, conformations, and dynamics of unentangled ionic PDMS melts, with cationic functionalizations either on the chain ends or randomly grafted along the backbone, using a united atom model and by means of atomistic molecular dynamics simulations. We found that the density of ionic PDMS melts increased in comparison to neutral PDMS melts. Moreover, the density of ionic melts increases with the fraction of charged monomers. The Br${}^{-}$ counterions are more strongly correlated and localized with the N${}^{+}$ cations in the end-functionalized chains, especially for the higher molecular weight systems, causing those chains to stretch. The Br${}^{-}$ counterions are also more strongly correlated to each other in the end-functionalized PDMS chains. This stronger condensation of Br${}^{-}$ to N${}^{+}$ leads to a slower diffusion of Br${}^{-}$ anions in the end-functionalized melts. In addition, the mobility of lower molecular weight ionic PDMS chains can enhance the Br${}^{-}$ diffusion. Neutral PDMS chains demonstrate the fastest diffusion, and the charged monomer fraction of ionic random PDMS copolymers enhances the Br${}^{-}$ diffusion, especially at higher temperatures; however, it also decreases the PDMS dynamics. Furthermore, while the end-chain functionalization of PDMS chains drastically hinders the Br${}^{-}$ diffusion, it has rather a subtle effect on PDMS diffusion. Finally, temperature has a weak but distinct effect on the Br${}^{-}$ counterions structure and also leads to an Arrhenius type of behavior for the Br${}^{-}$ counterions’ diffusion coefficient, with the activation energy depending on the ionic architecture. These findings contribute to our understanding on the correlations between structure and dynamical properties of ion-containing polymers.

## Figures and Tables

**Table 1:** Systems studied at T=300 K, 375 K, 425 K, 450 K, and 473 K. The mass of Br${}^{-}$ is about 80 g/mol, the excess mass of a N${}^{+}$-carrying side chain is ≈100 g/mol, the mass of a monomer is 74 g/mol.

System	*f*	*N*	Chains	Br${}^{-}$ Anions	Molecular Weight $M_{w}$
neutral		40	128	-	3055 g/mol
		80	128	-	6095 g/mol
end-functionalized	5%	40	128	256	3277 g/mol
	2.5%	80	128	256	6268 g/mol
randomly functionalized	10%	40	128	512	3474 g/mol
	10%	80	128	1024	6782 g/mol
	5%	40	128	256	3314 g/mol
	2.5%	80	128	256	6256 g/mol

## Data Availability

The data presented in this study are available in this article.

## Supplementary Materials

The following supporting information can be downloaded at: https://www.mdpi.com/article/10.3390/polym14153070/s1, Figure S1: Mean square displacement of Br${}^{-}$ anions; Figure S2: Mean square displacement of ionically functionalized PDMS melts. Figure S3: Apparent transference number.
