# Supplementary material for: Structure and Diffusion of Ionic PDMS Melts
Source: Polymers (Basel). 2022 Jul 29;14(15):3070. doi: 10.3390/polym14153070 (PMC9370667; doi:10.3390/polym14153070)
Supplement: Supplementary file 1 [file polymers-14-03070-s001.zip › SI.pdf]

Supplementary information

Morphology and diffusion of ionic PDMS melts

Argyrios V. Karatrantos,[*,†] Jettawat Khantaveramongkol,[†] and Martin Kröger[*,‡]

†*Materials Research and Technology, Luxembourg Institute of Science and Technology, 5, Avenue des Hauts-Fourneaux, L-4362 Esch-sur-Alzette, Luxembourg*

‡*Polymer Physics, Department of Materials, ETH Zurich, Leopold-Ruzicka-Weg 4, CH-8093 Zurich, Switzerland*

E-mail: argyrios.karatrantos@list.lu; mk@mat.ethz.ch

Contents

S1 Mean square displacement of Br anions

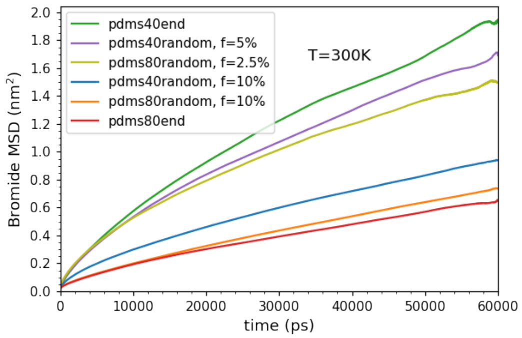

(a)

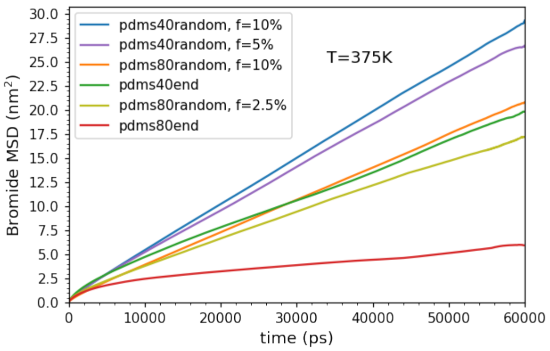

(b)

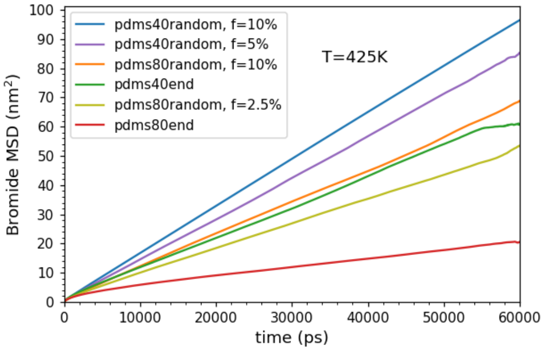

(c)

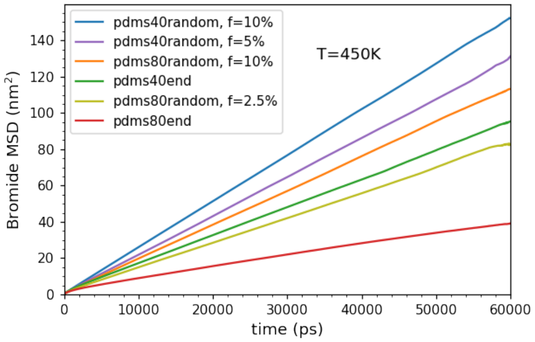

(d)

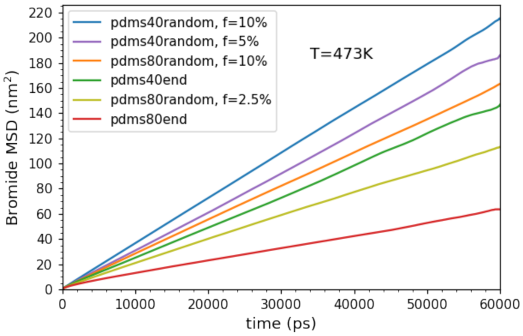

(e)

Figure S1: Mean square displacement versus time of Br^{-} anions in differently functionalized ionic PDMS melts, at **(a)** $T = 300$ K, **(b)** $T = 375$ K, **(c)** $T = 425$ K, **(d)** $T = 450$ K, and **(e)** $T = 473$ K

S2 Mean square displacement of PDMS chains

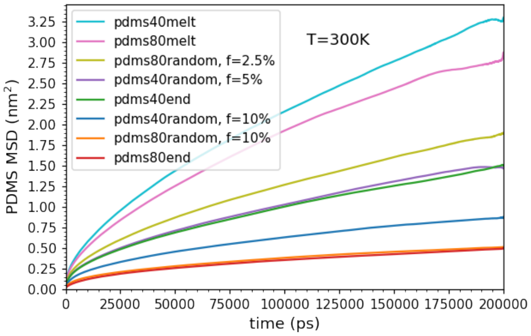

(a)

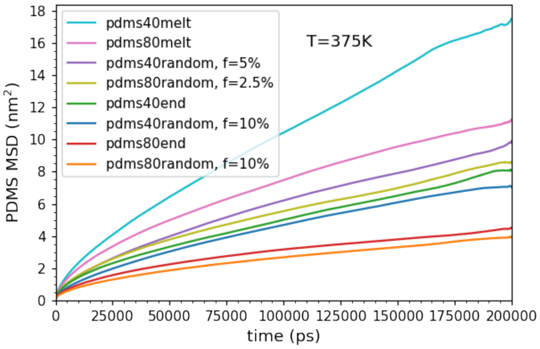

(b)

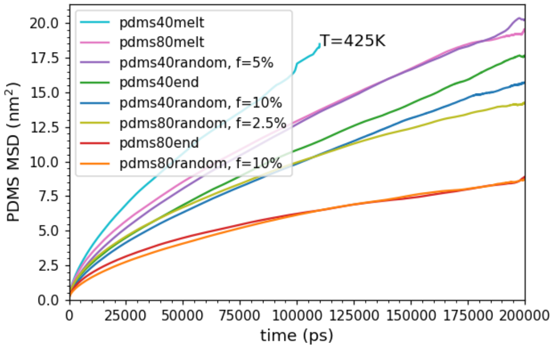

(c)

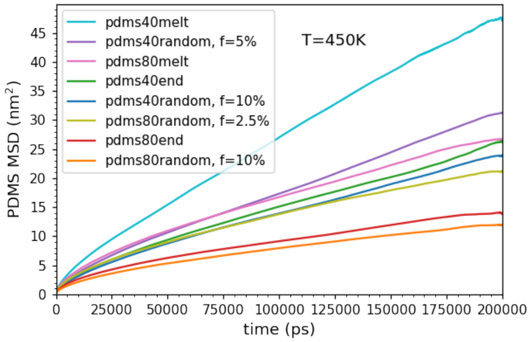

(d)

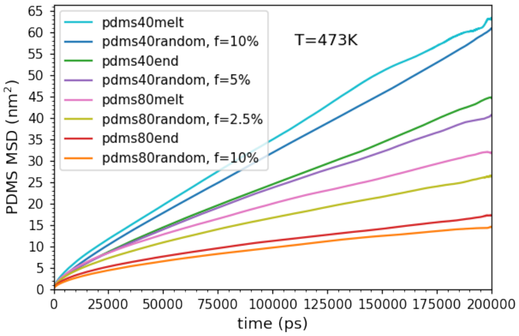

(e)

Figure S2: Mean square displacement versus time for differently functionalized PDMS melts at **(a)** $T = 300$ K, **(b)** $T = 375$ K, **(c)** $T = 425$ K **(d)** $T = 450$ K, and **(e)** $T = 473$ K.

S3 Apparent transference number

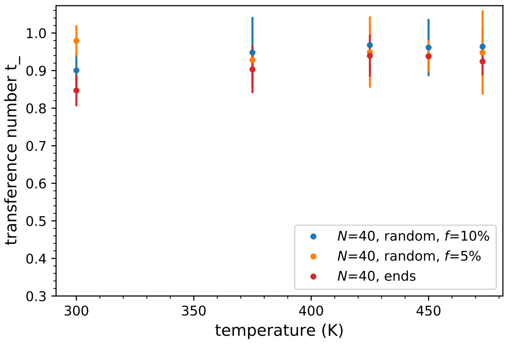

(a)

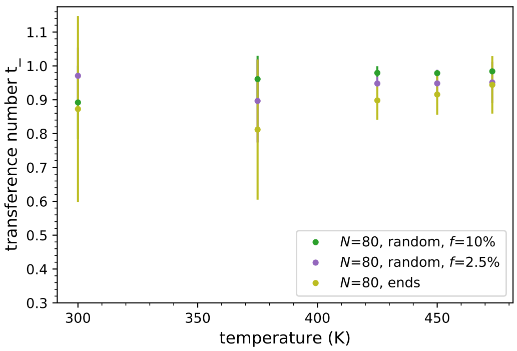

(b)

Figure S3: Apparent transference number t_- versus temperature T of PDMS chains end- and randomly grafted functionalized PDMS chains,**(a)** $N = 40$ K, **(b)** $N = 80$ K.
